# Cultural familiarity and musical expertise impact the pleasantness of consonance/dissonance but not its perceived tension

**DOI:** 10.1038/s41598-020-65615-8

**Published:** 2020-05-26

**Authors:** Imre Lahdelma, Tuomas Eerola

**Affiliations:** Durham University, Music Department, Durham, DH1 3RL United Kingdom

**Keywords:** Human behaviour, Perception

## Abstract

The contrast between consonance and dissonance is vital in making music emotionally meaningful. Consonance typically denotes perceived agreeableness and stability, while dissonance disagreeableness and a need of resolution. This study addresses the perception of consonance/dissonance in single intervals and chords with two empirical experiments conducted online. Experiment 1 explored the perception of a representative sample of intervals and chords to investigate the overlap between the seven most used concepts (Consonance, Smoothness, Purity, Harmoniousness, Tension, Pleasantness, Preference) denoting consonance/dissonance in all the available (60) empirical studies published since 1883. The results show that the concepts exhibit high correlations, albeit these are somewhat lower for non-musicians compared to musicians. In Experiment 2 the stimuli’s cultural familiarity was divided into three levels, and the correlations between the key concepts of Consonance, Tension, Harmoniousness, Pleasantness, and Preference were further examined. Cultural familiarity affected the correlations drastically across both musicians and non-musicians, but in different ways. Tension maintained relatively high correlations with Consonance across musical expertise and cultural familiarity levels, making it a useful concept for studies addressing both musicians and non-musicians. On the basis of the results a control for cultural familiarity and musical expertise is recommended for all studies investigating consonance/dissonance perception.

## Introduction

The origins of consonance and dissonance have been investigated since the days of Pythagoras in ancient Greece, and its elusive and mercurial nature baffles scholars to this day. The contrast between consonance and dissonance is a crucial feature of Western music, and it plays a vital role in making music emotionally meaningful by providing a sense of variety and motion^1–3^. Typically, consonant denotes connotations like *harmonious*, *agreeable*, and *stable*, while dissonant, in turn, connotations like *disagreeable*, *unpleasant*, and *in need of resolution*^4^. Consonance/dissonance has both a vertical and a horizontal aspect: single isolated intervals (two concurrent pitches) and chords (three or more concurrent pitches) represent *vertical consonance/dissonance*, while the sequential relationships between these in melodies and chord progressions represent *horizontal consonance/dissonance*^2^.

Aesthetic responses to consonance/dissonance (hereafter referred to as C/D and implying exclusively its vertical aspect) are surmised to have both biological and cultural roots, and the debate over which prevails represents a classical nature vs. nurture setting (e.g. ref. ^3^). In addition to disputes over its origins, also the very *definition* of C/D is notoriously problematic. As Tenney^5^ points out, “there is surely nothing in the language of discourse about music that is more burdened with purely semantic problems than are the terms *consonance and dissonance*” (p. 1). The concept itself is semantically loaded, and it has been volatile in a historical context as well: certain intervals (e.g., the major and minor thirds) became consonant only over time in the framework of Western music^6^. The inconsistencies arise not only from debates over which acoustic (e.g., roughness, harmonicity, fusion) and cultural phenomena (familiarity on both on a cultural and on an individual level, i.e., exposure) and their possible interactions might explain the underlying cause of C/D, but the term itself means different things to different scholars ranging from the most commonly associated definition *pleasantness* (e.g. ref. ^7^) to concepts like *preference* (e.g. ref. ^8^), *smoothness* (e.g. ref. ^9^), *clearness*^10^, *purity* (e.g. ref. ^11^), *tension* (e.g. ref. ^12^), and *harmoniousness* (e.g. ref. ^13^). While there have been a couple of attempts to compare the overlap between some of these associated concepts^12,14^, it is striking how most scholars do not problematise their definitions of C/D and take them at face value despite clear caveats in previous literature of automatically equating consonance with for example *pleasantness* or *preference*^15–18^. Moreover, Ritossa and Rickard^19^ suggest that *pleasantness* and *preference* are not directly linked concepts in music perception, yet these have been used as synonyms in C/D research by e.g., Bones *et al*.^7^, Prete *et al*.^20^, and McDermott *et al*.^21^.

The current study’s Experiment 1 aims to empirically explore the perception of the stimuli (single intervals and chords isolated from musical context) across those seven concepts (*Consonance*, *Smoothness*, *Purity*, *Harmoniousness*, *Tension*, *Pleasantness*, *Preference*) that have been most used to denote vertical C/D across all the available empirical studies reported since 1883 (in total 60). A related aim is to investigate the possible role of timbre in this by playing the stimuli with both the piano and the sine wave timbres as timbre can influence the perception of C/D^14^. Experiment 2 aims to further investigate the five key concepts (Consonance, Tension, Harmoniousness, Pleasantness, Preference) by addressing specific acoustic (roughness, harmonicity) and cultural (familiarity measured with the frequency of occurrence of the stimuli in actual music) contributors that might affect the perception of the stimuli across these. Moreover, both experiments aim to investigate the role of musical expertise in the perception of C/D as it has been suggested that the concepts of consonance and pleasantness correlate differently among musicians and non-musicians^14,22^. Also, both experiments will address the influence of the total number of pitches present in the stimuli (referred to as *numerosity*) on the ratings of C/D and related concepts as numerosity can affect the perception of C/D^23,24^.

## Experiment 1

### Methods

Experiment 1 is reported as one experiment but is actually a combination of seven separate sub-experiments. In each sub-experiment, participants rated through an online interface the stimuli on one of the seven concepts denoting C/D that have been most used in the previous empirical studies conducted since 1883. The review of past studies was carried out by the current authors and included exclusively those studies that used isolated, vertical pitch combinations (intervals and chords) as the experiment stimuli. The included studies were found with the aid of *Web of Science*, an online subscription-based scientific citation indexing service. The applied search terms were “*consonance dissonance*” (326 results), “*consonance perception*” (322 results), “*interval consonance*” (183 results), and “*chord consonance*” (125 results). As Web of Science keeps track of publications only from the year 1900 onwards, studies older than this were searched for manually.

The seven most common concepts to denote C/D are 1) *Pleasantness* (used in 31 studies), 2) *Consonance* (used in 15 studies), 3) *Smoothness* (used in 13 studies), 4) *Purity* (used in five studies), 5) *Harmoniousness* (used in four studies), 6) *Preference* (used in three studies), and 7) *Tension* (used in three studies). Those terms that evidently denote the same perceptual concept (e.g., antonyms like *smoothness/roughness*) were collapsed under one concept. The majority of the concepts have been used consistently during the 20th century and are in use to the present day. All of the concepts have been used to denote the perception of *both* intervals and chords; the concepts of *fusion* (used in eight studies), *beauty* (used in five studies), and *euphony* (used in five studies) were excluded as they have been used in studies involving exclusively intervals as the experiment stimuli. To minimise the effect of different interpretations of the concepts between participants, each one was explained on the basis of how the concepts are typically defined in previous research or in dictionary entries (see the Appendix). In the explanations, care was taken not to confound the pivotal concept of *Consonance* with the rest of the concepts.

#### Participants

As culture has been reported to affect the perception of C/D^21,25^, only Western participants (self-identified native English speakers) were recruited to avoid a cultural confound. The rationale behind choosing both musicians and non-musicians as participants was data-driven, as including both of these groups is the most common procedure (used in 26 studies) in the previous C/D studies. The participants were recruited through *Prolific Academic*, an online crowdsourcing platform targeted especially for research purposes. Previous research suggests that Prolific Academic participants consistently complete questionnaires carefully and the platform has high reliability^26,27^.

The participants’ musical expertise was measured with the six self-report rank items (*Which title best describes you?*) taken from the *Ollen Musical Sophistication Index*^28^. The six items were (1) *Non-musician*, (2) *Music-loving non-musician*, (3) *Amateur musician*, (4) *Serious amateur musician*, (5) *Semiprofessional musician*, and (6) *Professional musician*. Participants identifying themselves as belonging to groups 1–2 were categorised as “non-musicians”, while those belonging to groups 3–6 as “musicians”. For the benefits of using this strategy to assess musical expertise, see Zhang and Schubert^29^. In addition, participants’ age, gender, and music preference was assessed within the survey. The latter was divided into four meta-genres based on Rentfrow and Gosling^30^ by providing example genres as proxies for the four dimensions (*Reflective & Complex* - Classical/Ethnic, *Intense & Rebellious* - Rock/Heavy, *Upbeat & Conventional* - Pop/Electro, and *Energetic & Rhythmic* - Other). The participants were asked to choose one of these four genres to indicate their music preference. Informed consent was obtained from all participants. The experiment was approved by the ethics committee of the Department of Music at Durham University and was conducted in accordance with its guidelines and regulations.

The total amount of participants after removing outliers (see Procedure) was 407. The mean age of the participants was 35.04 (*SD* = 12.55, 57.2% females). Participants were randomly allocated to each sub-experiment from the overall pool in order to have a balanced sample of both musicians and non-musicians. This pool size was estimated on the basis of a previous experiment by Bowling *et al*.^31^ where thirty participants (15 musicians and 15 non-musicians) gave consonance ratings for all 12 dyads, 66 trichords, and 220 tetrachords (played with the piano timbre) that can be formed using the intervals specified by the chromatic scale over one octave. Our aim was to have twice the number of musicians and non-musicians in each concept to be able to evaluate the consistencies within the concepts reliably (see Supporting Information Table 1).

#### Materials

**Figure 1:**
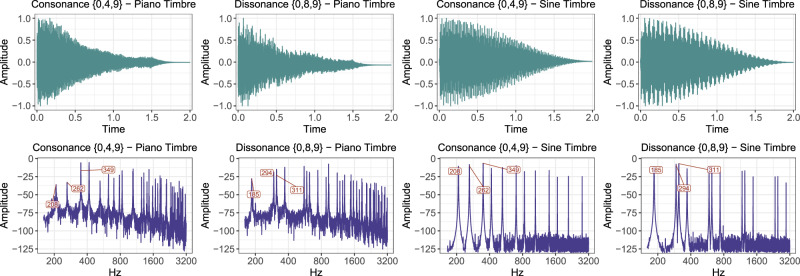
Two example stimuli (trichords representing consonance level extremes Q1 and Q5) played with the piano and sine wave timbres. The upper plot displays the waveform and the lower plot shows the frequency spectra with the *F*_0_ labelled in Hz for convenience.

For a representative continuum of C/D, the stimuli were chosen on the basis of the above-mentioned experiment conducted by Bowling *et al*.^31^ on the perception of C/D in intervals, trichords, and tetrachords. All intervals, trichords and tetrachords that were rank ordered according to perceived consonance by Bowling *et al*.^31^ were ordered into five quintiles of the mean consonance ratings. Out of these quintiles five intervals, 10 trichords, and 10 tetrachords were chosen in a randomised manner to represent a continuum of consonance, as two- (used in 42 studies), three- (used in 20 studies), and four-pitch (used 11 studies) combinations are the most used stimuli in the previous experiments conducted on C/D perception. Due to the smaller overall number of intervals than trichords and tetrachords, only one interval per quintile could be chosen to represent the respective consonance levels. With trichords and tetrachords there were always two chords representing a quintile of consonance. The total number of stimuli was thus 25 × 2 timbres = 50 (see Table 1). As per the procedure by Bowling *et al*.^31^, the fundamental frequencies (*F*_0_s) of the pitches in each interval and chord were adjusted so that the mean *F*_0_ of all pitches was C_4_ (261.63 Hz). The timbres used were the piano and sine wave, these being the two most commonly utilised timbres (the piano used in 18 studies, the sine wave in 14 studies) in the previous experiments conducted on C/D perception (see Fig. 1 for examples of the stimuli). The stimuli were played exclusively in equal temperament: again, this is the most common procedure in the previous C/D studies (used in 40 studies).

**Table 1:** The stimuli.

Consonance Level	Numerosity		
*Quantile boundaries*	*Intervals*	*Trichords*	*Tetrachords*
Q1 (diss.) 1.03–1.57	{0,1}	{0,8,9}, {0,3,11}	{0,1,2,8}, {0,1,7,9}
Q2 1.57–1.83	{0,10}	{0,6,7}, {0,2,3}	{0,1,4,9}, {0,3,4,9}
Q3 1.83–2.20	{0,6}	{0,4,5}, {0,7,10}	{0,2,8,11}, {0,2,3,8}
Q4 2.20–2.63	{0,8}	{0,5,10}, {0,3,10}	{0,4,8,12}, {0,3,9,12}
Q5 (cons.) 2.63–3.89	{0,12}	{0,7,12}, {0,4,9}	{0,5,7,12}, {0,5,7,9}

Quantile boundaries refer to the consonance ratings in Bowling *et al*.^31^ and the integer numbers are the pitches in each interval and chord.

The piano stimuli were generated with *Ableton Live 9* (a music sequencer software), using the *Synthogy Ivory Grand Pianos II* plug-in. The applied sound font was *Steinway D Concert Grand*. No reverb was used, and the intervals and chords had a fixed velocity (65) in order to have a neutral and even sound. The sine wave stimuli were generated with five partials with exponential decay in the successive amplitudes, ${a}_{n}={e}^{6-n}/{e}^{5}$. The temporal envelope of the sound was shaped with a half-Hanning window (duration of 2.0 seconds). All stimuli were normalised (to −3 db) with *Adobe Audition CC 2019* (a digital audio workstation) to control for any amplitude differences due to pitch numerosity and timbre dissimilarities. The sound files were converted to stereo (same signal in both channels) as 44.1 kHz, 32 bits per sample waveform audio files. These files were rendered as constant bit rate 320 kbps high quality stereo mp3 files for compatibility with the survey design software used in the experiment (see Procedure). The length of each interval and chord was exactly 2.0 seconds. The stimuli can be found online at https://osf.io/tupzq/.

#### Procedure

The online experiment was conducted with the *Qualtrics Survey Software*, a web-based survey tool. First, the participants’ demographic background data was collected (musical expertise, music preference, gender, age). Before the evaluation of the stimuli, the participants received written instructions and were asked to rate each interval and chord on the presented concept (see the Appendix). Each concept was rated on a Likert scale ranging from 1 to 5, the concepts’ bipolar extremes taken from previous research literature. With *Pleasantness*, the bipolar extremes were 1 = *Unpleasant* and 5 = *Pleasant* (e.g. ref. ^22^). With *Consonance*, the extremes were 1 = *Dissonant* and 5 = *Consonant* (e.g. ref. ^32^). With *Smoothness*, the extremes were 1 = *Rough* and 5 = *Smooth* (e.g. ref. ^12^). With *Purity*, the extremes were 1 = *Impure* and 5 = *Pure* (e.g. ref. ^33^). With *Harmoniousness*, the extremes were 1 = *Inharmonious* and 5 = *Harmonious* (e.g. ref. ^13^). With *Preference*, the extremes were 1 = *I don’t like it* and 5 = *I like it* (e.g. ref. ^20^). With *Tension*, the extremes were 1 = *Tense* and 5 = *Relaxed* (e.g. ref. ^14^). Participants were randomly allocated to one of the seven concept sub-experiments, and the order of the stimuli presentation was also randomised. All of the 50 separate pitch combinations were repeated once, resulting in 100 stimuli altogether. As there was a clear link between fast overall survey completion time and random response patterns, those participants (*n* = 58) who completed the experiment faster than the minimal time estimated for reasonable assessment (< 400 s overall, i.e., < 4 s/trial) were removed.

To summarise the experiment design, there are seven between-subject sub-experiments (one for each concept), all having the same stimuli (*n* = 100) broken down into four stimulus factors (*Consonance*: 5 levels, *Numerosity*: 3 levels, *Timbre*: 2 levels, *Repeat*: 2 levels) and four participant factors (*Musical Expertise*: 2 levels, *Music Preference*: 4 levels, *Gender*: 2 levels, *Age*).

### Results

The results will first focus on the concepts’ inter-rater reliability and their overall correlations and will then continue to the role of specific factors on the evaluations across the seven concepts. The internal consistencies of the concepts were measured with mean *r* correlation coefficients due to inflated values of the Cronbach alphas (*α*s > 0.93 for musicians, > 0.82 for non-musicians). Interestingly, by far the highest consistency among musicians was on the concept of *Harmoniousness* (0.52), followed by *Pleasantness* (0.45). For non-musicians the highest consistency was on the concept of *Tension* (0.30), followed by *Harmoniousness* (0.24). All in all, the consistencies were considerably higher for musicians than non-musicians (see Table 2).

**Table 2:** Correlations across the seven concepts for musicians and non-musicians (*df* = 98) and average correlations across the participants (reliability).

	Cons.	Smoothn.	Purity	Harmon.	Tension	Pleas.	Pref.
**Musicians**							
Smoothness	0.955						
Purity	0.950	0.954					
Harmoniousness	0.956	0.954	0.951				
Tension	−0.926	−0.947	−0.932	−0.937			
Pleasantness	0.959	0.949	0.933	0.951	−0.928		
Preference	0.921	0.937	0.914	0.938	−0.949	0.936	
*Reliability*	*0.362*	*0.421*	*0.389*	*0.519*	*0.360*	*0.447*	*0.293*
**Non-Musicians**							
Smoothness	0.800						
Purity	0.892	0.804					
Harmoniousness	0.870	0.885	0.847				
Tension	−0.862	−0.689↑*	−0.841	−0.818			
Pleasantness	0.866	0.745	0.830	0.850	−0.882		
Preference	0.777	0.502↑*	0.730	0.663↑*	−0.896	0.801	
*Reliability*	*0.197*	*0.145*	*0.136*	*0.239*	*0.295*	*0.183*	*0.236*

All correlations ≤ 0.05 with multiple correction. Significance values between correlations using Fisher’s *Z* tests where * ≤ 0.05.

As can be seen from the correlation table (Table 2) the coefficients between *Consonance* and the rest of the concepts were conspicuously high and consistent especially in the case of musicians (all correlations > 0.90). For non-musicians the correlations were somewhat lower, but also consistent (all correlations > 0.80, with the exception of *Preference*’s 0.78). The highest correlations with *Consonance* for musicians were on the concepts of *Pleasantness* (0.96), *Harmoniousness* (0.96), and *Smoothness* (0.96), while for non-musicians on the concepts of *Purity* (0.89), *Harmoniousness* (0.87), and *Pleasantness* (0.87). For both groups the lowest correlations with *Consonance* were on the concept of *Preference* (0.92 for musicians and 0.78 for non-musicians).

To explore the differences between the concepts and factors, first a repeated MANOVA was conducted across the seven concepts and the eight factors (Numerosity, Consonance Level, Repeat, Timbre, Expertise, Age, Gender, Music Preference) with the participants as random effects. Strong main effects for Concept (*df* = 403, *t* = 2.02, *p* ≤ 0.05), Numerosity (*df* = 40286, *t* = −9.62, *p* ≤ 0.001), Consonance Level (*df* = 40286, *t* = 40.82, *p* ≤ 0.001), Timbre (*df* = 40286, *t* = −2.09, *p* ≤ 0.05), and Expertise (*df* = 532.9, *t* = −3.171, *p* ≤ 0.01) were observed, but no significant main effects for Repeat (*df* = 401, *t* = −0.009, *p* = 0.993), Age (*df* = 401, *t* = 1.62, *p* = 0.106), Gender (*df* = 401, *t* = −1.90, *p* = 0.058), and Music Preference (*df* = 401, *t* = 0.30, *p* = 0.77).

A more detailed generalised linear mixed model (GLMM) analysis was carried out within each concept to better highlight the different ways the factors operated across the concepts. Table 3 shows the breakdown of the GLMM analyses across the seven concepts and four factors with the participants as random effects. To save space, only the estimates and the p values are shown for the main effects across the concepts. Supporting Information Tables 2–8 displays the full statistical table with interactions.

**Table 3:** GLMM estimates across the seven concepts and four factors.

Factor	Cons.	Pleas.	Smoothn.	Purity	Harmon.	Pref.	Tension
Numerosity	−0.186***	−0.074***	−0.202***	−0.233***	−0.193***	−0.079***	0.204***
Cons. Level	0.396***	0.384***	0.377***	0.358***	0.466***	0.337***	−0.388***
Timbre	−0.069*	−0.093***	0.058*	−0.133***	0.023	−0.513***	0.386***
Expertise	0.089	−0.145	0.033	−0.341*	−0.119	0.142	−0.034

*p ≤ 0.05, **p ≤ 0.01, ***p ≤ 0.001.

**Figure 2:**
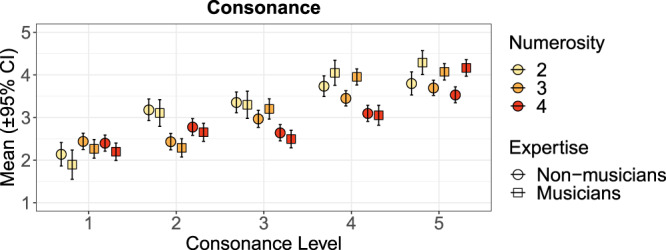
Consonance ratings across Consonance Level, Numerosity, and Expertise.

Figure 2 summarises the ratings for one concept (Consonance) and the three most important factors (Consonance Level, Numerosity, and Expertise) as an example. The complete breakdown across different factor combinations can be seen from Supporting Information Figs. 1, 2, and 3.

#### Numerosity

Numerosity affected all seven concepts. On the concept of *Consonance* the intervals were perceived as more consonant, except in the case of the most dissonant sonorities. This tendency was exactly the same for the concept of *Purity*. All in all, higher numerosity created more perceived dissonance, roughness, impurity, inharmoniousness, and tension especially on the middle level of C/D in the stimuli. Notably, this was not mirrored in perceived *Pleasantness* and *Preference*, where higher numerosity yielded slightly higher ratings across various levels of C/D (see Supporting Information Fig. 2).

#### Consonance

In all seven sub-experiments, the Consonance Level showed a significant effect across the five levels (see Table 3 for statistical significance, and also see Supporting Information Fig. 1 for the full pattern). Ratings typically increased from dissonant to consonant levels in a linear fashion (reverse for *Tension*).

#### Timbre

All of the concepts were affected by timbre statistically significantly, with the exception of *Harmoniousness*. The sine wave timbre was generally perceived as more dissonant, unpleasant, impure, and tense, and it was preferred less than the piano timbre. The difference between the two timbres was especially conspicuous on the concepts of *Tension* and *Preference*. However, on the concept of *Smoothness* this pattern was broken, where the most consonant intervals and tetrachords as well as the most dissonant trichords and tetrachords were perceived slightly smoother when played on the sine wave timbre (see Supporting Information Fig. 2).

#### Expertise

None of the concepts were affected by musical expertise statistically significantly with the exception of *Purity*, where non-musicians perceived the more dissonant stimuli as noticeably purer than musicians (see Supporting Information Fig. 3). This implies that for non-musicians, consonance and purity are not completely overlapping concepts when the stimuli are highly dissonant.

### Discussion

It is striking how high and consistent the correlations between the seven concepts were especially for musically trained participants. For musically less-trained participants the correlations were somewhat lower, but also consistent. The only notable exception was the concept of *Preference* which had a somewhat lower correlation (0.78) in the case of non-musicians. The results imply that both groups – especially musicians – have virtually a blueprint of an acoustic concept (vertical consonance and dissonance) that they rate similarly across semantically quite distantly related concepts (e.g., purity vs. pleasantness). It is worth noting that the concept of *Preference* had the lowest correlation with *Consonance* across both musicians and non-musicians and showed by far the lowest internal consistency in the case of musicians; this raises concerns about its validity to reliably measure the perception of consonance.

With regard to different factors, higher numerosity typically resulted in higher perceived dissonance, roughness, impurity, inharmoniousness, and tension especially on the middle level of C/D in the stimuli. This is notably in line with the notion that the addition of pitches to a chord typically increases its *roughness*^23,24^, an acoustic component seen as prevalent in dissonant, but not in consonant musical chords^34^. However, the current results imply that higher pitch numerosity does not automatically result in a lack of preference and pleasantness despite a higher amount of perceived dissonance. On the contrary, it seems to increase ratings of pleasantness and preference in the case of consonant chords; this finding is line with previous research on the perception of isolated chords^35^.

In terms of timbre, the sine wave sound was typically perceived as more dissonant, unpleasant, impure, and tense, and it was preferred less than the piano. A plausible explanation for this is that the sine wave sound is simply less familiar than the common piano sound. The difference according to timbre was especially prominent on the concepts of *Tension* and *Preference* where the piano timbre was perceived less tense and was also preferred more. This finding is line with previous research conducted with isolated chords where both perceived preference^36^ and pleasantness^14^ were affected by timbre. Interestingly, in the current study timbre did not affect the concept of *Harmoniousness*. This implies that using this particular concept may have advantages in C/D research when multiple timbres are involved; it also exhibited good inter-rater reliability across both musicians and non-musicians.

## Experiment 2

As Experiment 1 was concerned only with representing a seamless continuum of C/D without addressing specific acoustic or cultural contributors, the question of cultural familiarity was not yet investigated. There is a consensus that the overall perception of C/D in Western sonorities is presumably based on a combination of *roughness*, *harmonicity*, and *familiarity* (e.g. refs. ^2,37^). Roughness denotes the sound quality that arises from the beating of frequency components (e.g. refs. ^10,34^), and harmonicity indicates how closely a sonority’s spectrum corresponds to a harmonic series (e.g. ref. ^38^). The order of importance between these two acoustic factors on the perception of C/D is debated^37^. In addition to the acoustic phenomena of roughness and harmonicity, exposure (i.e., familiarity on both on a cultural and on an individual level) has been surmised to be an essential contributor to perceived C/D^17,39^, and its important role has been empirically demonstrated both in the case of intervals^40^ and chords^41,42^. As cultural familiarity is evidently an important factor in C/D perception, the current experiment quantifies the stimuli’s cultural familiarity with the aid of a corpus-based familiarity model by Harrison and Pearce^37^. As explained by Harrison and Pearce, their model is based on the hypothesis that listeners become familiar with vertical pitch combinations in proportion to their frequency of occurrence in the listener’s musical culture, and that this familiarity positively influences consonance through the mere exposure effect^37^. Their model simulates a Western listener’s musical exposure by counting the frequencies of occurrence of different vertical pitch combinations in the *Billboard Data Set*^43^, a large corpus of music sampled from the US charts published between 1958 and 1991.

### Methods

As with Experiment 1, Experiment 2 was conducted online and is reported as one experiment but consists of five separate sub-experiments. The key concepts of *Consonance*, *Tension*, *Harmoniousness*, *Pleasantness*, and *Preference* were further investigated. *Consonance* was included as the benchmark against which the other concepts were measured. *Tension* was added as it had a high negative correlation with *Consonance* and good internal consistency across participants with varying musical expertise in Experiment 1. Moreover, tension has been found to correlate strongly with perceived dissonance in previous research on the perception of isolated chords^35^. *Harmoniousness* was included as it too showed a high correlation with *Consonance* and good internal consistency across both musicians and non-musicians in Experiment 1. *Pleasantness* was included as it is the most used concept in the previous empirical experiments on C/D perception, and *Preference* was of interest as it was the concept that correlated least with *Consonance* in Experiment 1, regardless of participants’ musical expertise.

#### Participants

The criteria for inclusion and the recruitment procedure was identical to Experiment 1. Informed consent was obtained from all participants. The experiment was approved by the ethics committee of the Department of Music at Durham University and was conducted in accordance with its guidelines and regulations. The total amount of participants after removing participants below the overall duration threshold (*n* = 102) was 510. The size of the participant pool was estimated so that there were at least 30 musicians and 30 non-musicians in each sub-experiment to be comparable with Bowling *et al*.^31^ and also with the current study’s Experiment 1. To balance the musical expertise across the concepts, we randomly sampled musicians and non-musicians for each of the five concepts, resulting in a total of 392 participants (60% females). The mean age of the participants was 33.92 (*SD* = 12.61). The number of participants was 80 in the *Consonance*, *Tension*, and *Preference* sub-experiments, 78 in the *Pleasantness* sub-experiment, and 74 in the *Harmoniousness* sub-experiment (for a breakdown according to musical expertise, see Supporting Information Table 1).

#### Materials

As with Experiment 1, the stimuli were chosen from all the empirically rank-ordered intervals, trichords, and tetrachords according to perceived C/D by Bowling *et al*.^31^. The stimuli were randomised to represent three respective levels of cultural familiarity (frequency), quantified with the previously introduced model by Harrison and Pearce^37^: (1) High, (2) In-between, and (3) Low. The number of stimuli per one familiarity level was always 24, making the total number of stimuli 3 × 24 = 72. The stimuli were created with an identical procedure as in Experiment 1, with the exception that only the piano timbre was used to keep the experiment within a reasonable time frame for the participants. The stimuli can be found online at https://osf.io/tupzq/.

#### Procedure

The procedure for the experiment was identical to Experiment 1 apart from that each pitch combination (72 in total) was evaluated only once in order to avoid making the experiment too long due to the large number of stimuli. Participants were allocated to one of the five sub-experiments until the pre-determined number of participants in each sub-experiment (see Participants) was complete. As with Experiment 1, there was a clear correlation between fast overall survey completion time and random response patterns; removing those participants who completed the experiment in less than 280 seconds provided an effective filter for outliers.

The experiment design consisted of five between-subject sub-experiments (one for each concept), all having the same stimuli (*n* = 72) broken down into two stimulus factors (*Familiarity*: 3 levels, *Numerosity*: 3 levels), and four participant factors (*Musical Expertise*: 2 levels, *Music Preference*: 4 levels, *Gender*: 2 levels, and *Age*).

### Results

In the results first the concepts’ inter-rater reliability and the overall correlations will be reported, then moving to the role of specific factors on the evaluations across the five concepts. The internal consistencies of the concepts were measured with mean r correlation coefficients. The highest consistency for musicians was on the concept of *Consonance* (0.36), while for non-musicians on the concept of *Pleasantness* (0.27). The consistencies were again higher for musicians than non-musicians (see Table 4), as in the case of Experiment 1.

**Table 4:** Correlations across the five concepts and three levels of familiarity for musicians and non-musicians (*df* = 70) and correlations among the participants (reliability).

	Consonance	Tension	Pleasantness	Preference	Harmoniousness
**Musicians: Familiarity Level 1**					
Tension	−0.851				
Pleasantness	0.703↑*	−0.865			
Preference	0.647	−0.744	0.870		
Harmoniousness	0.891↑**	−0.750	0.778	0.771	
**Musicians: Familiarity Level 2**					
Tension	−0.746				
Pleasantness	0.451↑**	−0.545			
Preference	0.605	−0.731	0.851		
Harmoniousness	0.766	−0.489↑*	0.671↑**	0.664	
**Musicians: Familiarity Level 3**					
Tension	−0.951				
Pleasantness	0.960	−0.929			
Preference	0.952	−0.929	0.975		
Harmoniousness	0.982↑**	−0.927	0.968	0.967	
*Reliability (all levels)*	*0.355*	*0.241*	*0.330*	*0.289*	*0.225*
**Non-musicians: Familiarity Level 1**					
Tension	−0.809				
Pleasantness	0.824	−0.851			
Preference	0.578↑**	−0.680↑*	0.766		
Harmoniousness	0.737	−0.837↑*	0.869	0.620	
**Non-musicians: Familiarity Level 2**					
Tension	−0.722				
Pleasantness	0.818	−0.797			
Preference	0.731	−0.676	0.760		
Harmoniousness	0.493↑*	−0.552	0.768	0.535	
**Non-musicians: Familiarity Level 3**					
Tension	−0.954				
Pleasantness	0.953	−0.952			
Preference	0.943	−0.942	0.925		
Harmoniousness	0.947	−0.936	0.968	0.924	
*Reliability (all levels)*	*0.157*	*0.171*	*0.207*	*0.267*	*0.166*

All correlations ≤ 0.05 with multiple correction within Familiarity Levels. Significance values between correlations using Fisher’s *Z* tests where * ≤ 0.05, and ** ≤ 0.01.

As can be seen from the correlation table (Table 4) the correlations between *Consonance* and the rest of the concepts were drastically affected by familiarity across both musicians and non-musicians. Remarkably, for musicians the concepts that transcended all three levels of familiarity in terms of retaining relatively high correlations with *Consonance* were *Harmoniousness* (Level 1 = 0.89, Level 2 = 0.77, Level 3 = 0.98) and *Tension* (Level 1 = −0.85, Level 2 = −0.75, Level 3 = −0.95), while for non-musicians it was *Pleasantness* (Level 1 = 0.82, Level 2 = 0.82, Level 3 = 0.95) and *Tension* (Level 1 = −0.81, Level 2 = −0.72, Level 3 = −0.95).

Notably, for musicians the correlation between *Consonance* and *Pleasantness* was somewhat low (0.70) when the stimuli were familiar (Level 1), but it dropped even lower (0.45, *Z* = 2.24, *p *≤ 0.05) on the in-between level (Level 2). In the case of non-musicians it is noteworthy how the correlations between *Consonance* and *Pleasantness* and *Consonance* and *Preference* show very different patterns: while *Pleasantness* had a high correlation with *Consonance* consistently, the correlation between *Preference* and *Consonance* was surprisingly low (0.58) when the stimuli were familiar (Level 1). This difference in correlations between *Consonance* and *Pleasantness* and *Consonance* and *Preference* is statistically significant (*Z* = 3.00, *p *≤ 0.005, see Table 4). Overall, the correlation between *Consonance* and *Preference* was low across both musicians and non-musicians when the stimuli were familiar. It is also of particular interest that unfamiliar stimuli (Level 3) yielded extremely high correlations (> 0.90) between all concepts irrespective of musical expertise, echoing the results of Experiment 1.

To explore the differences between the concepts and factors, first a repeated MANOVA was conducted across the five concepts and the six factors (Numerosity, Familiarity Level, Expertise, Age, Gender, Music Preference) with the participants as random effects. Strong main effects for Numerosity (*df* = 27828, *t* = −2.927, *p* ≤ 0.01), Familiarity Level (*df* = 27828, *t* = −4.384, *p* ≤ 0.001), and Expertise (*df* = 729.5, *t* = 2.573, *p* ≤ 0.05) were observed, but no significant main effects for Age (*df* = 386, *t* = 1.14, *p* = 0.26), Gender (*df* = 386, *t* = −0.08, *p* = 0.93), and Music Preference (*df* = 386, *t* = 1.00, *p* = 0.32).

A more detailed generalised linear mixed model (GLMM) analysis was carried out within each concept to investigate how the factors operated across the concepts. Table 5 shows the breakdown of the GLMM analyses across the five concepts and three factors with the participants as random effects. To save space, only the estimates and the p values are shown for the main effects across the concepts (for the full table, see Supporting Information Tables 9–13).

**Table 5:** GLMM estimates across the five concepts and three factors.

Factor	Consonance	Tension	Harmoniousness	Pleasantness	Preference
Familiarity Level	−0.483***	0.429***	−0.565***	−0.464***	−0.426***
Numerosity	−0.320***	0.242***	−0.299**	−0.157*	−0.118
Expertise	0.084	0.217*	0.052	0.123	0.026

*p ≤ 0.05, **p ≤ 0.01, ***p ≤ 0.001.

#### Familiarity

**Figure 3:**
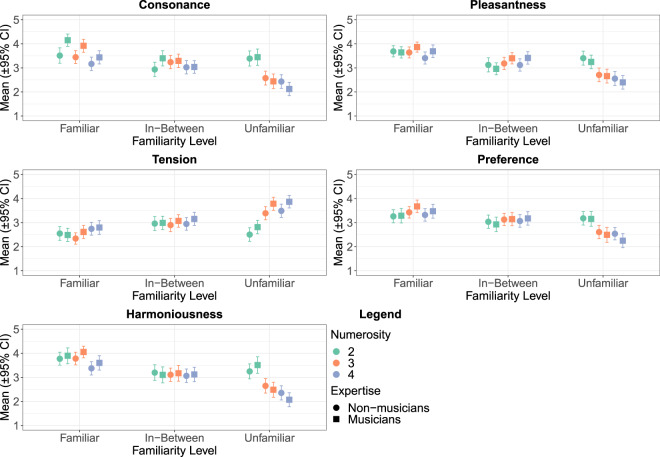
Ratings on the five concepts across Familiarity Level, Numerosity, and Expertise.

Cultural familiarity had a strong effect on all of the five concepts. The general tendency was that with chords (trichords and tetrachords) the concepts of *Consonance*, *Harmoniousness*, *Pleasantness*, and *Preference* exhibited a pattern of decreasing means from familiar (Level 1), through in-between (Level 2) to unfamiliar (Level 3). This tendency was inverted in the case of *Tension*. Curiously, intervals did not follow this same pattern (see Fig. 3).

#### Numerosity

Numerosity affected all of the scales with the exception of *Preference*. On the scale of *Consonance* the familiar (Level 1) and unfamiliar (Level 3) intervals were perceived as more consonant than the chords. Notably, this was not mirrored in the *Preference* ratings when the intervals were familiar (Level 1). Unfamiliar (Level 3) intervals were clearly perceived as more relaxed, harmonious, and pleasant when compared to the chords (see Fig. 3).

#### Expertise

The only concept that was affected by musical expertise was *Tension*. Musicians perceived the stimuli consistently more tense than non-musicians and this tendency was especially prominent when the stimuli were unfamiliar (Level 3). The only exception to this trend were the familiar (Level 1) intervals which non-musicians perceived as slightly more tense than musicians (see Fig. 3).

#### Role of key stimulus features

As the overall perception of C/D in Western sonorities is surmised to be based on a combination of *roughness*, *harmonicity*, and *familiarity* (e.g. refs. ^2,37^), the correlations between these key stimulus features and the five concepts were investigated across both musicians and non-musicians with the data of Experiment 2. The theoretical roughness values of the stimuli were assessed with the model of Hutchinson and Knopoff^34^; this model has been demonstrated to be the best predictor of C/D ratings among several roughness models as it had the highest partial correlation with average consonance ratings in the Bowling *et al*.^31^ dataset according to a recent meta analysis^37^. Harmonicity was assessed with a model by Harrison and Pearce^44^ which simulates the way listeners search the auditory spectrum for occurrences of harmonic spectra, and cultural familiarity was measured with the already introduced (see Experiment 2) corpus-based familiarity model by Harrison and Pearce^37^.

Roughness correlates negatively with all concepts except *Tension* (*r*(70) = −0.630, 0.527, −0.590, −0.454, −0.434 for *Consonance*, *Tension*, *Harmoniousness*, *Pleasantness*, and *Preference*, respectively, all *p *≤ 0.001) and these correlations were only somewhat impacted by the other two acoustic variables when we look at the semi-partial correlations (−0.376, 0.281, −0.417, −0.327, and −0.334). However, harmonicity, which is positively correlated with the ratings except for *Tension* (0.478, −0.422, 0.384, 0.280, and 0.245, all *p *≤ 0.05), carries only negligible independent contribution after controlling for roughness and cultural familiarity (−0.037, −0.009, −0.138, −0.148, −0.180). Familiarity is positively correlated with the ratings apart from *Tension* (0.609, −0.629, 0.647, 0.664, 0.684, all *p *≤ 0.001) and seems to be independent of both roughness and harmonicity since the semi-partial correlations remain roughly at the same level (0.490, −0.530, 0.548, 0.594, 0.622).

### Discussion

The results suggest that cultural familiarity drastically influences the perception of C/D in isolated pitch combinations. The data demonstrates a curious dualism in this: in the case of musicians the concepts that correlate most with *Consonance* while transcending all three levels of cultural familiarity are *Harmoniousness* and *Tension*, while for non-musicians they are *Pleasantness* and *Tension*. In other words, cultural familiarity strongly affects the perception of C/D for both musicians and non-musicians, but in noticeably different ways. The current results suggest that this tendency is not directly related to music preferences (cf. Popescu *et al*.^22^). Music preferences might nonetheless act as indicators of different amounts of exposure to varying prevalence of pitch combinations within distinct musical styles. Strikingly, the only concept that transcends both expertise and familiarity levels is *Tension*: its correlations with *Consonance* were always > 0.70, regardless of musical expertise and cultural familiarity.

With regard to different factors, higher numerosity typically yielded more perceived dissonance, replicating the results of Experiment 1. Notably, however, this was again not mirrored on the concept of *Preference* where chords with higher numerosity and hence acoustic roughness were preferred more across both musicians and non-musicians when the chords were either familiar or on the in-between level. In terms of musical expertise, the only concept affected was *Tension* where musicians generally perceived the stimuli as more tense than non-musicians.

On the level of specific concepts, the perception of *Consonance* seems to be more related to acoustic roughness whereas the concepts of *Preference* and *Pleasantness* have higher correlations with cultural familiarity; this finding is in line with previous research linking familiarity with preference^45^. It is noteworthy how Terhardt^46^ also suggests that the consonance evaluation of isolated chords is dominated by the concept of *sensory consonance* (i.e., lack of roughness). The current findings corroborate Terhardt’s view, but notably cultural familiarity’s role seems to be even more important than acoustic roughness when the individual stimulus features’ independent contributions are examined.

## Conclusions and Future Research

In previous research literature the exact relationship between *Consonance*, *Pleasantness*, and *Preference* has been notoriously contentious, some scholars arguing against equating between these concepts (e.g. refs. ^15,25^) while others not problematising this association to any extent (e.g. refs. ^7,21,37^). This evidently simplifying direct linkage is endorsed by even such authoritative sources as Grove Music Online^47^. The current findings show that these two starkly opposing views can be integrated when using cultural familiarity as a quantified variable, and when taking into account the role of musical expertise.

The results suggest that musicians conceive *Pleasantness* and *Consonance* as distinct concepts, as opposed to non-musicians, and this finding is in line with Arthurs *et al*.^14^, Popescu *et al*.^22^, and van de Geer *et al*.^12^. Arguably this is due to musicians being more familiar with different pitch combinations overall due to musical training: for musicians the correlation between *Consonance* and *Pleasantness* dropped conspicuously only when the stimuli were culturally familiar or moderately familiar. Moreover, it is possible that musicians are also more accustomed to the terminology of C/D, resulting in a more finely grained vocabulary for describing interval and chord perception which leads to the distinction between the concepts of *Consonance* and *Pleasantness*. However, also for musically less trained participants the correlation between *Consonance* and *Preference* was much lower than between *Consonance* and *Pleasantness* when the stimuli were culturally familiar, suggesting a dissimilarity between these two concepts. This finding is notably in line with research where actual musical excerpts were used^19^ and has important implications for C/D research, as for example Bones *et al*.^7^, Prete *et al*.^20^, and McDermott *et al*.^21^ have been using pleasantness and preference as synonyms in studies investigating the perception of isolated intervals and chords. The current results clearly demonstrate the poor consistency of the concept of *Preference* and its low correlation with *Consonance* across both musicians and non-musicians when the stimuli are culturally familiar. Hence, we suggest for future research to clearly distinguish between the concepts of *Pleasantness* and *Preference* and recommend avoiding *Preference* as a synonym for consonance altogether.

The findings strongly suggest that *Tension* is the best concept to use as a synonym for C/D when both musicians and non-musicians are included as it retained relatively high correlations with *Consonance* across all levels of cultural familiarity. The concept of *Pleasantness* is a valid choice for non-musicians and is usable across various levels of cultural familiarity but should not be implemented in studies involving musicians, unless the stimuli are completely unfamiliar culturally. The concept of *Harmoniousness* might have advantages when multiple timbres are involved and when the stimuli are culturally unfamiliar; in this setting it may be used across both musicians and non-musicians. In terms of numerosity it may be concluded that while higher numerosity typically results in more perceived dissonance, roughness, impurity, inharmoniousness, and tension, this does not automatically result in a lack of preference when the stimuli are relatively consonant or culturally familiar.

With the aid of modern-day computing power and big corpus data, quantifying the elusive aspect of cultural familiarity has become possible and the current study demonstrates its crucial role in the perception of C/D among Western listeners. On the basis of the results more research is suggested on identifying the best possible acoustic predictors and especially their interactions in addition to the cultural familiarity aspect to further explain their role in the perception of vertical consonance/dissonance.

## Supplementary information

Appendix.

Supplementary Figures and Tables.

## Data Availability

The complete data set for this study, including the stimuli can be accessed online at the Open Science Framework public repository (https://osf.io/tupzq/).

## References

[1] Meyer, L. B. *Emotion and Meaning in Music* (Chicago: Chicago University Press, 1956).

[2] Parncutt R, Hair G (2011). Consonance and dissonance in music theory and psychology: Disentangling dissonant dichotomies. J. Interdiscip. Music. Stud..

[3] Perlovsky, L. I. *Music: Passions and Cognitive Functions* (San Diego, CA: Elsevier, 2017).

[4] Tramo MJ, Cariani PA, Delgutte B, Braida LD (2001). Neurobiological foundations for the theory of harmony in Western tonal music. Annals New York Acad. Sci..

[5] Tenney, J. *A History of ‘Consonance’ and ‘Dissonance’* (New York: Excelsior Music Publishing Company, 1988).

[6] Hindemith, P. *The Craft of Musical Composition*, Vol. 1 (New York: Belwin-Mills, 1942).

[7] Bones O, Hopkins K, Krishnan A, Plack CJ (2014). Phase locked neural activity in the human brainstem predicts preference for musical consonance. Neuropsychologia.

[8] Butler JW, Daston PG (1968). Musical consonance as musical preference: A cross-cultural study. The J. Gen. Psychol..

[9] Roberts L (1986). Consonance judgements of musical chords by musicians and untrained listeners. Acustica.

[10] Kameoka A, Kuriyagawa M (1969). Consonance theory part I: Consonance of dyads. The J. Acoust. Soc. Am..

[11] Rasmussen, M., Santurette, S. & MacDonald, E. Consonance perception of complex-tone dyads and chords. In *Proceedings of the Seventh Forum Acusticum* (European Acoustics Association, 2014).

[12] van de Geer J, Levelt W, Plomp R (1962). The connotation of musical consonance. Acta Psychol..

[13] Cook ND (2001). Explaining harmony: The roles of interval dissonance and chordal tension. Annals New York Acad. Sci..

[14] Arthurs Y, Beeston AV, Timmers R (2018). Perception of isolated chords: Examining frequency of occurrence, instrumental timbre, acoustic descriptors and musical training. Psychol. Music.

[15] Cazden Norman (1972). The Systemic Reference of Musical Consonance Response. International Review of the Aesthetics and Sociology of Music.

[16] Malmberg CF (1918). The perception of consonance and dissonance. Psychol. Monogr..

[17] Parncutt R (2006). Commentary on Cook & Fujisawa’s “The psychophysics of harmony perception: Harmony is a three-tone phenomenon”. Empir. Music. Rev..

[18] Valentine C (1914). The method of comparison in experiments with musical intervals and the effect of practice on the appreciation of discords. Br. J. Psychol..

[19] Ritossa DA, Rickard NS (2004). The relative utility of ‘pleasantness’ and ‘liking’ dimensions in predicting the emotions expressed by music. Psychol. Music.

[20] Prete, G., Fabri, M., Foschi, N., Brancucci, A. & Tommasi, L. The “consonance effect” and the hemispheres: A study on a split-brain patient. *Laterality: Asymmetries Body. Brain Cogn.* **20**, 257–269 (2015). 10.1080/1357650X.2014.959525 25256169

[21] McDermott JH, Schultz AF, Undurraga EA, Godoy RA (2016). Indifference to dissonance in native Amazonians reveals cultural variation in music perception. Nature.

[22] Popescu, T. *et al*. The pleasantness of sensory dissonance is mediated by musical style and expertise. *Sci. Reports* **9** (2019). 10.1038/s41598-018-35873-8 PMC6355932 30705379

[23] Bowling DL, Purves D (2015). A biological rationale for musical consonance. Proc. Natl. Acad. Sci..

[24] Lahdelma, I., Armitage, J. & Eerola, T. Affective priming with musical chords is influenced by pitch numerosity. *Music. Sci*., 10.1177/1029864920911127 (2020).

[25] Maher TF (1976). “Need for resolution” ratings for harmonic musical intervals: A Comparison between Indians and Canadians. J. Cross-Cultural Psychol..

[26] Palan S, Schitter C (2018). Prolific.ac—a subject pool for online experiments. J. Behav. Exp. Finance.

[27] Peer E, Brandimarte L, Samat S, Acquisti A (2017). Beyond the Turk: Alternative platforms for crowdsourcing behavioral research. J. Exp. Soc. Psychol..

[28] Ollen, J. E. A criterion-related validity test of selected indicators of musical sophistication using expert ratings. Ph.D. thesis, The Ohio State University (2006).

[29] Zhang JD, Schubert E (2019). A single item measure for identifying musician and nonmusician categories based on measures of musical sophistication. Music Percept..

[30] Rentfrow, P. J. & Gosling, S. D. The do re mi’s of everyday life: The structure and personality correlates of music preferences. *J. Pers. Soc. Psychol.* **84**, 1236–1256 (2003). 10.1037/0022-3514.84.6.1236 12793587

[31] Bowling DL, Purves D, Gill KZ (2018). Vocal similarity predicts the relative attraction of musical chords. Proc. Natl. Acad. Sci..

[32] Plomp R, Levelt WJM (1965). Tonal consonance and critical bandwidth. The J. Acoust. Soc. Am..

[33] Vos J (1986). Purity ratings of tempered fifths and major thirds. Music Percept..

[34] Hutchinson W, Knopoff L (1978). The acoustic component of Western consonance. Interface.

[35] Lahdelma I, Eerola T (2016). Mild dissonance preferred over consonance in single chord perception. i-Perception.

[36] Lahdelma I, Eerola T (2016). Single chords convey distinct emotional qualities to both naive and expert listeners. Psychol. Music.

[37] Harrison P, Pearce M (2020). Simultaneous consonance in music perception and composition. Psychol. Rev..

[38] Parncutt, R. *Harmony: A Psychoacoustical Approach* (Berlin: Springer-Verlag, 1989).

[39] Cazden N (1980). The definition of consonance and dissonance. Int. Rev. Aesthet. Sociol. Music.

[40] Weiss Michael W., Cirelli Laura K., McDermott Josh H., Trehub Sandra E. (2020). Development of consonance preferences in Western listeners. Journal of Experimental Psychology: General.

[41] Lahdelma, I. At the interface between sensation and emotion: Perceived qualities of single chords. *Ph.D. thesis, University of Jyväskylä* (2017).

[42] McLachlan N, Marco D, Light M, Wilson S (2013). Consonance and pitch. J. Exp. Psychol. Gen..

[43] Burgoyne, J. A. Stochastic processes and database-driven musicology. *Ph.D. thesis, McGill University* (2011).

[44] Harrison, P. & Pearce, M. An energy-based generative sequence model for testing sensory theories of Western harmony. In *Proceedings of the 19th International Society for Music Information Retrieval Conference*, 160–167 (Paris, France, 2018).

[45] Zajonc R (2001). Mere exposure: A gateway to the subliminal. Curr. Dir. Psychol. Sci..

[46] Terhardt E (1984). The concept of musical consonance: A link between music and psychoacoustics. Music Percept..

[47] Palisca, C. V. & Moore, B. C. *Consonance. Grove Music Online* (2001).

