# Supplementary material for: Cultural familiarity and musical expertise impact the pleasantness of consonance/dissonance but not its perceived tension
Source: Sci Rep. 2020 May 26;10:8693. doi: 10.1038/s41598-020-65615-8 (PMC7250829; doi:10.1038/s41598-020-65615-8)
Supplement: Supplementary file 1 — Appendix. [file 41598_2020_65615_MOESM1_ESM.pdf]

Appendix

Instructions

In this experiment you will be asked to listen to different kinds of sound combinations and evaluate them on a 5-point scale on the shown adjective. You will also be asked to provide some information concerning your musical education and demographic background. You can listen to each sound combination as many times as you like before evaluating it. Each sound combination should be evaluated as a separate entity, regardless of preceding or sequential ones. We strongly recommend using headphones for listening.

Definition of Each Consonance Concept

The consonance concepts were defined and explained to the participants in the experiments as follows:

1. **Pleasantness**. How pleasant do you think the sound combination is?
2. **Consonance**. How consonant do you think the sound combination is? Note that this is a purely subjective question in sound perception: as an adjective "consonance" usually denotes things like "agreement" and "compatibility".*
3. **Smoothness**. How smooth do you think the sound combination is?
4. **Purity**. How pure do you think the sound combination is?
5. **Harmoniousness**. How harmonious do you think the sound combination is? Note that this is a purely subjective question in sound perception: as an adjective "harmonious" usually means that elements are working well together.*
6. **Preference**. How much do you like the sound combination?
7. **Tension**. How tense do you think the sound combination is?

*The additional explanations/synonyms for the concepts of *consonance* and *harmoniousness* were taken from the Oxford and Cambridge dictionaries.
