# Supplementary material for: Cultural familiarity and musical expertise impact the pleasantness of consonance/dissonance but not its perceived tension
Source: Sci Rep. 2020 May 26;10:8693. doi: 10.1038/s41598-020-65615-8 (PMC7250829; doi:10.1038/s41598-020-65615-8)
Supplement: Supplementary file 2 — Supplementary Figures and Tables. [file 41598_2020_65615_MOESM2_ESM.pdf]

# 1 Supporting Information

Additional figures and tables for study titled "Cultural familiarity and musical expertise impact the pleasantness of consonance/dissonance but not its perceived tension" by Lahdelma, I. & Eerola, T. in 2020, published in *Scientific Reports*, https://doi.org/10.1038/s41598-020-65615-8.

## 1.1 Figures

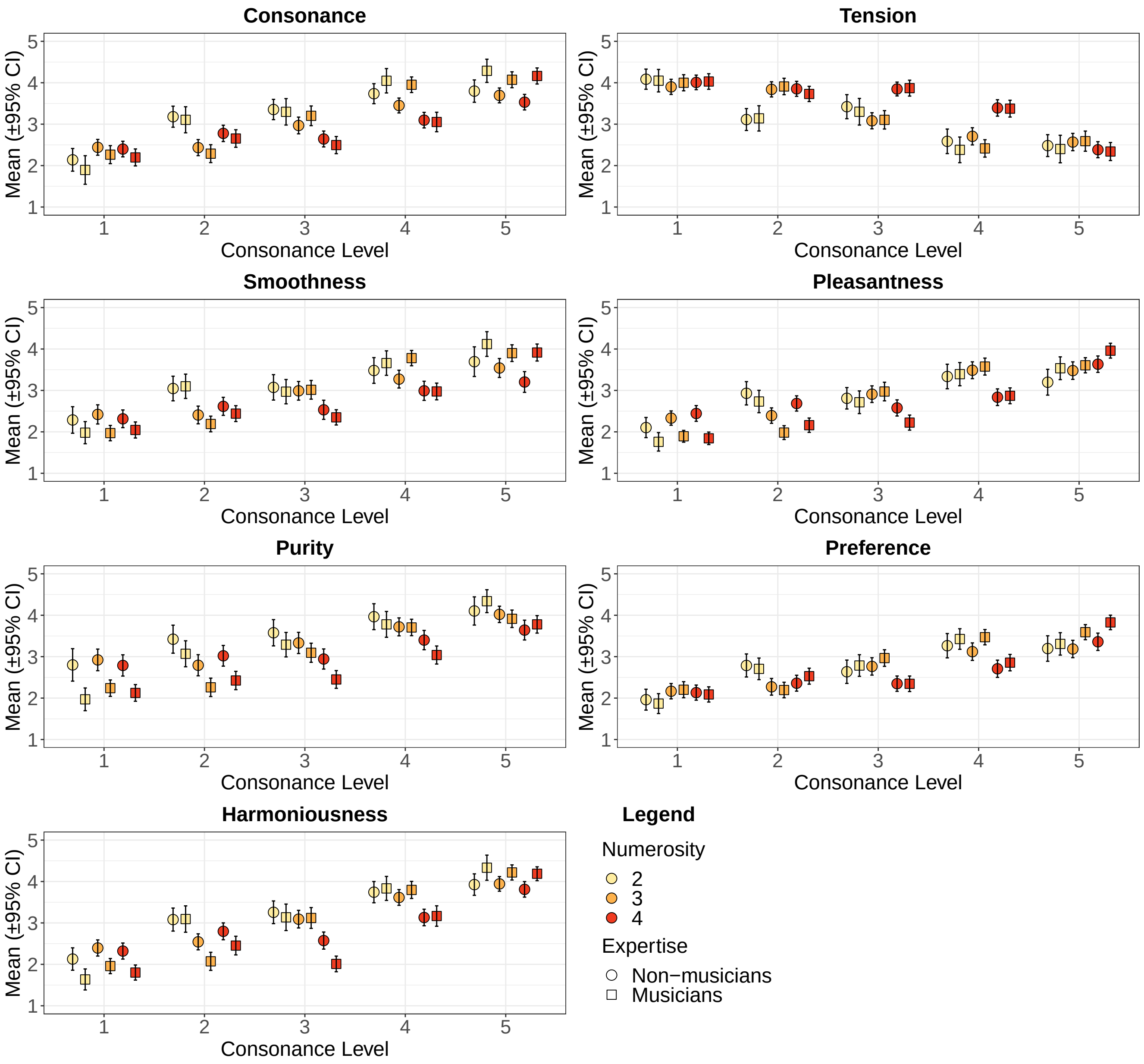


**Figure 1.** Ratings of all concepts across Consonance Level, Numerosity, and Musical Expertise.

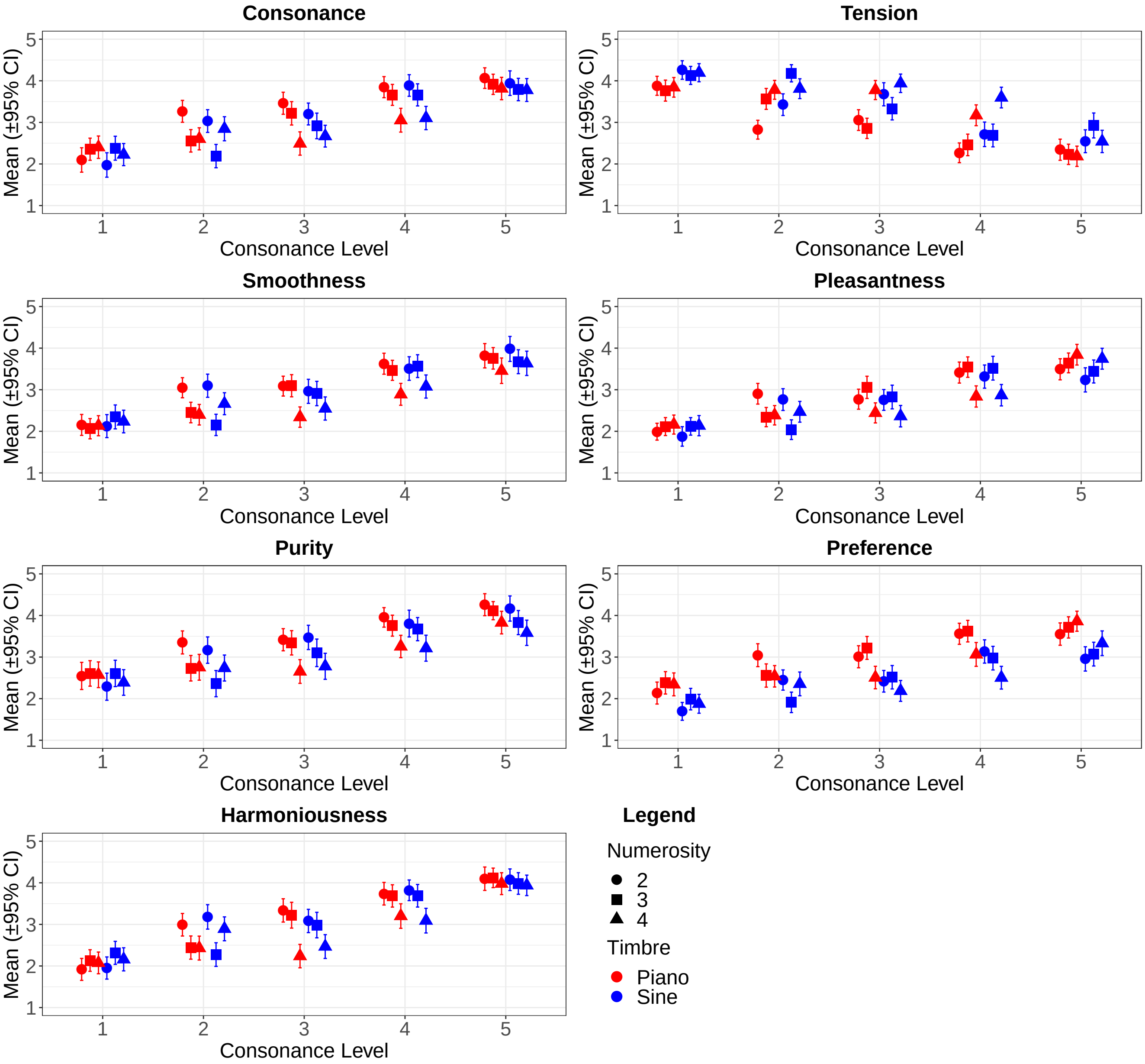


**Figure 2.** Ratings of all concepts across Consonance Level, Numerosity, and Timbre.

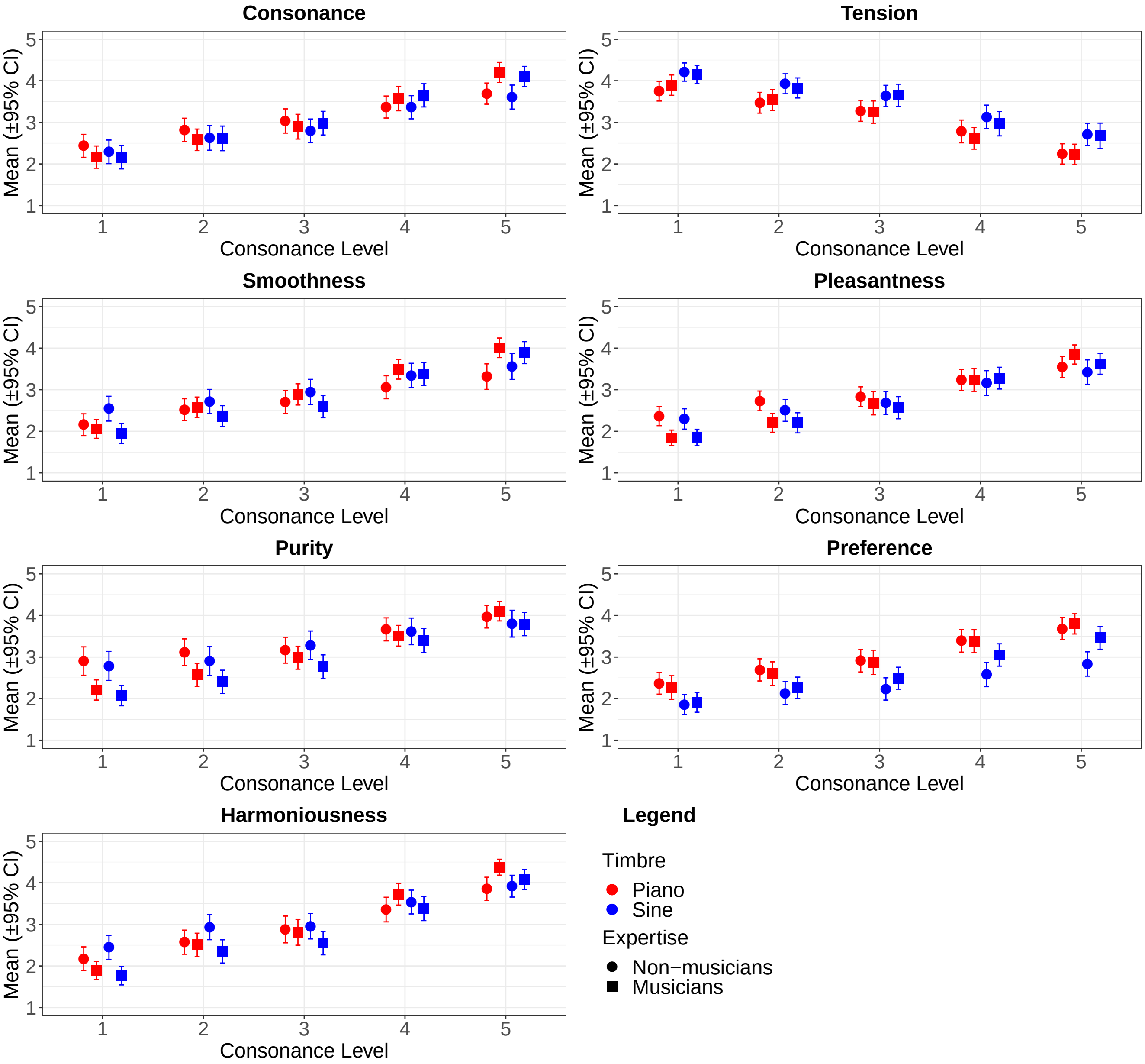


**Figure 3.** Ratings of all concepts across Consonance Level, Timbre, and Musical Expertise.

## 1.2 Tables

| Concept | Experiment 1 | Experiment 2 |
|---|---|---|
| | N (Mus./Non-mus.) | N (Mus./Non-mus.) |
| Consonance | 62 (26/36) | 80 (40/40) |
| Tension | 54 (25/29) | 80 (40/40) |
| Pleasantness | 57 (28/29) | 78 (38/40) |
| Preference | 67 (34/33) | 80 (40/40) |
| Harmoniousness | 61 (24/37) | 74 (34/40) |
| Smoothness | 52 (25/27) | NA |
| Purity | 54 (25/29) | NA |
| *Total* | 407 | 392 |

**Table 1.** Participant numbers and expertise across the concepts and experiments.

| Factor | Estimate | Std. Error | df | t value | P value |
|---|---|---|---|---|---|
| Intercept | 2.12 | 0.427 | 139 | 4.97 | 1.91e-06 |
| Numerosity | -0.0437 | 0.0811 | 5628 | -0.539 | 0.59 |
| Cons. Level | 0.451 | 0.0804 | 5628 | 5.61 | 2.15e-08 |
| Repeat | 0.628 | 0.377 | 5628 | 1.67 | 0.0956 |
| Timbre | -0.0406 | 0.377 | 5628 | -0.108 | 0.914 |
| Expertise | -0.151 | 0.12 | 52 | -1.26 | 0.213 |
| Gender | -0.113 | 0.13 | 52 | -0.867 | 0.39 |
| Genre | -0.00292 | 0.0728 | 52 | -0.0402 | 0.968 |
| Age | 0.00155 | 0.00531 | 52 | 0.292 | 0.772 |
| Numerosity×Cons. Level | -0.0152 | 0.0245 | 5628 | -0.62 | 0.535 |
| Numerosity×Repeat | -0.165 | 0.115 | 5628 | -1.44 | 0.151 |
| Cons. Level×Repeat | -0.157 | 0.114 | 5628 | -1.38 | 0.168 |
| Numerosity×Timbre | 0.012 | 0.115 | 5628 | 0.105 | 0.916 |
| Cons. Level×Timbre | -0.0536 | 0.114 | 5628 | -0.472 | 0.637 |
| Repeat×Timbre | -0.389 | 0.533 | 5628 | -0.731 | 0.465 |
| Numerosity×Cons. Level×Repeat | 0.0434 | 0.0346 | 5628 | 1.25 | 0.21 |
| Numerosity×Cons. Level×Timbre | 0.0113 | 0.0346 | 5628 | 0.326 | 0.744 |
| Numerosity×Repeat×Timbre | 0.0991 | 0.162 | 5628 | 0.611 | 0.541 |
| Cons. Level×Repeat×Timbre | 0.0632 | 0.161 | 5628 | 0.393 | 0.694 |
| Numerosity×Cons. Level×Repeat×Timbre | -0.0202 | 0.0489 | 5628 | -0.413 | 0.68 |

**Table 2.** GLMM results for Pleasantness across main factors and their interactions with participants as random factors. Four background factors have been also included in the analysis although the interactions have not been included in the GLMM analysis.

| Factor | Estimate | Std. Error | df | t value | P value |
|---|---|---|---|---|---|
| Intercept | 1.44 | 0.421 | 196 | 3.41 | 0.00078 |
| Numerosity | 0.0512 | 0.0873 | 6123 | 0.586 | 0.558 |
| Cons. Level | 0.665 | 0.0865 | 6123 | 7.69 | 1.72e-14 |
| Repeat | 0.527 | 0.406 | 6123 | 1.3 | 0.194 |
| Timbre | -0.384 | 0.406 | 6123 | -0.947 | 0.344 |
| Expertise | 0.0992 | 0.109 | 57 | 0.913 | 0.365 |
| Gender | -0.0703 | 0.109 | 57 | -0.647 | 0.52 |
| Genre | 0.0518 | 0.0658 | 57 | 0.787 | 0.435 |
| Age | 0.00608 | 0.00472 | 57 | 1.29 | 0.203 |
| Numerosity×Cons. Level | -0.088 | 0.0263 | 6123 | -3.34 | 0.000832 |
| Numerosity×Repeat | -0.182 | 0.123 | 6123 | -1.48 | 0.14 |
| Cons. Level×Repeat | -0.121 | 0.122 | 6123 | -0.989 | 0.323 |
| Numerosity×Timbre | 0.0853 | 0.123 | 6123 | 0.69 | 0.49 |
| Cons. Level×Timbre | -0.0141 | 0.122 | 6123 | -0.115 | 0.909 |
| Repeat×Timbre | -0.294 | 0.574 | 6123 | -0.512 | 0.609 |
| Numerosity×Cons. Level×Repeat | 0.0419 | 0.0372 | 6123 | 1.13 | 0.26 |
| Numerosity×Cons. Level×Timbre | 0.00853 | 0.0372 | 6123 | 0.229 | 0.819 |
| Numerosity×Repeat×Timbre | 0.096 | 0.175 | 6123 | 0.55 | 0.583 |
| Cons. Level×Repeat×Timbre | 0.0995 | 0.173 | 6123 | 0.575 | 0.565 |
| Numerosity×Cons. Level×Repeat×Timbre | -0.0319 | 0.0527 | 6123 | -0.606 | 0.544 |

**Table 3.** GLMM results for Consonance. The main factors and their interactions with participants as random factors. Four background factors have been also included in the analysis although the interactions have not been included in the GLMM analysis.

| Factor | Estimate | Std. Error | df | t value | P value |
|---|---|---|---|---|---|
| Intercept | 1.86 | 0.507 | 114 | 3.67 | 0.000371 |
| Numerosity | -0.102 | 0.0926 | 5133 | -1.1 | 0.271 |
| Cons. Level | 0.536 | 0.0917 | 5133 | 5.84 | 5.53e-09 |
| Repeat | 0.123 | 0.43 | 5133 | 0.287 | 0.774 |
| Timbre | -0.602 | 0.43 | 5133 | -1.4 | 0.162 |
| Expertise | 0.00378 | 0.128 | 47 | 0.0295 | 0.977 |
| Gender | -0.065 | 0.13 | 47 | -0.502 | 0.618 |
| Genre | 0.122 | 0.0779 | 47 | 1.57 | 0.124 |
| Age | 0.000307 | 0.00445 | 47 | 0.0691 | 0.945 |
| Numerosity×Cons. Level | -0.0431 | 0.0279 | 5133 | -1.55 | 0.122 |
| Numerosity×Repeat | -0.0122 | 0.131 | 5133 | -0.0934 | 0.926 |
| Cons. Level×Repeat | 0.014 | 0.13 | 5133 | 0.108 | 0.914 |
| Numerosity×Timbre | 0.189 | 0.131 | 5133 | 1.44 | 0.149 |
| Cons. Level×Timbre | 0.104 | 0.13 | 5133 | 0.801 | 0.423 |
| Repeat×Timbre | 0.598 | 0.608 | 5133 | 0.983 | 0.326 |
| Numerosity×Cons. Level×Repeat | -0.0158 | 0.0395 | 5133 | -0.4 | 0.689 |
| Numerosity×Cons. Level×Timbre | -0.0298 | 0.0395 | 5133 | -0.755 | 0.45 |
| Numerosity×Repeat×Timbre | -0.142 | 0.185 | 5133 | -0.765 | 0.444 |
| Cons. Level×Repeat×Timbre | -0.217 | 0.183 | 5133 | -1.18 | 0.237 |
| Numerosity×Cons. Level×Repeat×Timbre | 0.0589 | 0.0558 | 5133 | 1.06 | 0.291 |

**Table 4.** GLMM results for Smoothness. The main factors and their interactions with participants as random factors. Four background factors have been also included in the analysis although the interactions have not been included in the GLMM analysis.

| Factor | Estimate | Std. Error | df | t value | P value |
|---|---|---|---|---|---|
| Intercept | 2.76 | 0.554 | 98.3 | 4.99 | 2.66e-06 |
| Numerosity | -0.0446 | 0.0916 | 5331 | -0.486 | 0.627 |
| Cons. Level | 0.557 | 0.0908 | 5331 | 6.13 | 9.46e-10 |
| Repeat | 0.196 | 0.426 | 5331 | 0.461 | 0.645 |
| Timbre | -0.451 | 0.426 | 5331 | -1.06 | 0.29 |
| Expertise | -0.317 | 0.167 | 49 | -1.9 | 0.0637 |
| Gender | -0.176 | 0.176 | 49 | -1 | 0.322 |
| Genre | -0.112 | 0.0986 | 49 | -1.14 | 0.26 |
| Age | 0.0142 | 0.0065 | 49 | 2.19 | 0.0333 |
| Numerosity×Cons. Level | -0.061 | 0.0276 | 5331 | -2.21 | 0.0274 |
| Numerosity×Repeat | -0.0676 | 0.13 | 5331 | -0.522 | 0.602 |
| Cons. Level×Repeat | -0.0143 | 0.128 | 5331 | -0.111 | 0.911 |
| Numerosity×Timbre | 0.11 | 0.13 | 5331 | 0.846 | 0.398 |
| Cons. Level×Timbre | 0.103 | 0.128 | 5331 | 0.805 | 0.421 |
| Repeat×Timbre | 0.0532 | 0.602 | 5331 | 0.0883 | 0.93 |
| Numerosity×Cons. Level×Repeat | 0.00516 | 0.0391 | 5331 | 0.132 | 0.895 |
| Numerosity×Cons. Level×Timbre | -0.0345 | 0.0391 | 5331 | -0.883 | 0.377 |
| Numerosity×Repeat×Timbre | -0.019 | 0.183 | 5331 | -0.104 | 0.917 |
| Cons. Level×Repeat×Timbre | -0.0987 | 0.182 | 5331 | -0.543 | 0.587 |
| Numerosity×Cons. Level×Repeat×Timbre | 0.0291 | 0.0553 | 5331 | 0.527 | 0.599 |

**Table 5.** GLMM results for Purity. The main factors and their interactions with participants as random factors. Four background factors have been also included in the analysis although the interactions have not been included in the GLMM analysis.

| Factor | Estimate | Std. Error | df | t value | P value |
|---|---|---|---|---|---|
| Intercept | 2.32 | 0.416 | 212 | 5.59 | 7.05e-08 |
| Numerosity | -0.179 | 0.0885 | 6024 | -2.02 | 0.0429 |
| Cons. Level | 0.525 | 0.0877 | 6024 | 5.98 | 2.31e-09 |
| Repeat | -0.304 | 0.411 | 6024 | -0.74 | 0.459 |
| Timbre | -0.354 | 0.411 | 6024 | -0.86 | 0.39 |
| Expertise | -0.108 | 0.11 | 56 | -0.978 | 0.332 |
| Gender | 0.0784 | 0.109 | 56 | 0.721 | 0.474 |
| Genre | -0.164 | 0.0752 | 56 | -2.18 | 0.0332 |
| Age | 0.00455 | 0.00407 | 56 | 1.12 | 0.268 |
| Numerosity×Cons. Level | -0.0115 | 0.0267 | 6024 | -0.43 | 0.667 |
| Numerosity×Repeat | 0.0816 | 0.125 | 6024 | 0.652 | 0.514 |
| Cons. Level×Repeat | 0.141 | 0.124 | 6024 | 1.14 | 0.256 |
| Numerosity×Timbre | 0.153 | 0.125 | 6024 | 1.22 | 0.222 |
| Cons. Level×Timbre | 0.0859 | 0.124 | 6024 | 0.693 | 0.488 |
| Repeat×Timbre | -0.0166 | 0.582 | 6024 | -0.0286 | 0.977 |
| Numerosity×Cons. Level×Repeat | -0.0402 | 0.0377 | 6024 | -1.06 | 0.287 |
| Numerosity×Cons. Level×Timbre | -0.0403 | 0.0377 | 6024 | -1.07 | 0.286 |
| Numerosity×Repeat×Timbre | 0.0407 | 0.177 | 6024 | 0.23 | 0.818 |
| Cons. Level×Repeat×Timbre | -0.085 | 0.175 | 6024 | -0.485 | 0.628 |
| Numerosity×Cons. Level×Repeat×Timbre | 0.0183 | 0.0534 | 6024 | 0.342 | 0.732 |

**Table 6.** GLMM results for Harmoniousness. The main factors and their interactions with participants as random factors. Four background factors have been also included in the analysis although the interactions have not been included in the GLMM analysis.

| Factor | Estimate | Std. Error | df | t value | P value |
|---|---|---|---|---|---|
| Intercept | 2.19 | 0.431 | 146 | 5.09 | 1.11e-06 |
| Numerosity | -0.0744 | 0.0776 | 6618 | -0.959 | 0.337 |
| Cons. Level | 0.37 | 0.0769 | 6618 | 4.81 | 1.54e-06 |
| Repeat | 0.423 | 0.36 | 6618 | 1.17 | 0.241 |
| Timbre | -0.521 | 0.36 | 6618 | -1.44 | 0.149 |
| Expertise | 0.09 | 0.125 | 62 | 0.718 | 0.476 |
| Gender | -0.272 | 0.128 | 62 | -2.13 | 0.0371 |
| Genre | 0.141 | 0.0781 | 62 | 1.81 | 0.0754 |
| Age | -0.000747 | 0.00499 | 62 | -0.15 | 0.882 |
| Numerosity×Cons. Level | -0.00341 | 0.0234 | 6618 | -0.146 | 0.884 |
| Numerosity×Repeat | -0.128 | 0.11 | 6618 | -1.16 | 0.244 |
| Cons. Level×Repeat | -0.0648 | 0.109 | 6618 | -0.596 | 0.551 |
| Numerosity×Timbre | 0.039 | 0.11 | 6618 | 0.356 | 0.722 |
| Cons. Level×Timbre | -0.0239 | 0.109 | 6618 | -0.22 | 0.826 |
| Repeat×Timbre | -0.526 | 0.51 | 6618 | -1.03 | 0.302 |
| Numerosity×Cons. Level×Repeat | 0.0203 | 0.0331 | 6618 | 0.612 | 0.54 |
| Numerosity×Cons. Level×Timbre | -0.00299 | 0.0331 | 6618 | -0.0903 | 0.928 |
| Numerosity×Repeat×Timbre | 0.172 | 0.155 | 6618 | 1.11 | 0.268 |
| Cons. Level×Repeat×Timbre | 0.0608 | 0.154 | 6618 | 0.395 | 0.693 |
| Numerosity×Cons. Level×Repeat×Timbre | -0.0251 | 0.0468 | 6618 | -0.536 | 0.592 |

**Table 7.** GLMM results for Preference. The main factors and their interactions with participants as random factors. Four background factors have been also included in the analysis although the interactions have not been included in the GLMM analysis.

| Factor | Estimate | Std. Error | df | t value | P value |
|---|---|---|---|---|---|
| Intercept | 3.71 | 0.411 | 170 | 9.01 | 4.16e-16 |
| Numerosity | 0.224 | 0.0855 | 5331 | 2.62 | 0.00882 |
| Cons. Level | -0.442 | 0.0847 | 5331 | -5.22 | 1.89e-07 |
| Repeat | -0.393 | 0.397 | 5331 | -0.99 | 0.322 |
| Timbre | 0.725 | 0.397 | 5331 | 1.82 | 0.0683 |
| Expertise | -0.0364 | 0.105 | 49 | -0.345 | 0.731 |
| Gender | -0.0326 | 0.112 | 49 | -0.291 | 0.772 |
| Genre | -0.0145 | 0.0728 | 49 | -0.2 | 0.843 |
| Age | 0.000133 | 0.00423 | 49 | 0.0313 | 0.975 |
| Numerosity×Cons. Level | 0.0135 | 0.0258 | 5331 | 0.523 | 0.601 |
| Numerosity×Repeat | 0.124 | 0.121 | 5331 | 1.03 | 0.305 |
| Cons. Level×Repeat | 0.15 | 0.12 | 5331 | 1.25 | 0.211 |
| Numerosity×Timbre | -0.124 | 0.121 | 5331 | -1.03 | 0.305 |
| Cons. Level×Timbre | 0.0407 | 0.12 | 5331 | 0.34 | 0.734 |
| Repeat×Timbre | 0.713 | 0.562 | 5331 | 1.27 | 0.204 |
| Numerosity×Cons. Level×Repeat | -0.0454 | 0.0365 | 5331 | -1.24 | 0.214 |
| Numerosity×Cons. Level×Timbre | -0.00648 | 0.0365 | 5331 | -0.178 | 0.859 |
| Numerosity×Repeat×Timbre | -0.218 | 0.171 | 5331 | -1.27 | 0.203 |
| Cons. Level×Repeat×Timbre | -0.312 | 0.169 | 5331 | -1.84 | 0.0658 |
| Numerosity×Cons. Level×Repeat×Timbre | 0.0955 | 0.0516 | 5331 | 1.85 | 0.0641 |

**Table 8.** GLMM results for Tension. The main factors and their interactions with participants as random factors. Four background factors have been also included in the analysis although the interactions have not been included in the GLMM analysis.

| Concept and Factor | Estimate | Std. Error | df | t value | P value |
|---|---|---|---|---|---|
| Intercept | 2.35 | 0.765 | 195 | 3.08 | 0.00239 |
| Fam. Level | 0.209 | 0.304 | 160 | 0.688 | 0.493 |
| Numerosity | 0.172 | 0.277 | 160 | 0.619 | 0.537 |
| Expertise | 1.08 | 0.3 | 3364 | 3.61 | 0.000316 |
| Gender | -0.024 | 0.0944 | 75 | -0.254 | 0.8 |
| Genre | 0.0195 | 0.0458 | 75 | 0.426 | 0.671 |
| Age | 0.00904 | 0.00379 | 75 | 2.39 | 0.0196 |
| Fam. Level × Numerosity | -0.105 | 0.122 | 160 | -0.862 | 0.39 |
| Fam. Level × Expertise | -0.302 | 0.12 | 5606 | -2.53 | 0.0115 |
| Numerosity × Expertise | -0.186 | 0.109 | 5606 | -1.71 | 0.0878 |
| Fam. Level × Numerosity × Expertise | 0.00337 | 0.0481 | 5606 | 0.0701 | 0.944 |

**Table 9.** GLMM results for Consonance. The main factors and their interactions with participants as random factors. Four background factors have been also included in the analysis although the interactions with the main experimental factors have not been included in the GLMM analysis.

| Factor | Estimate | Std. Error | df | t value | P value |
|---|---|---|---|---|---|
| Intercept | 4.05 | 0.737 | 218 | 5.5 | 1.06e-07 |
| Fam. Level | -0.097 | 0.272 | 154 | -0.357 | 0.722 |
| Numerosity | -0.186 | 0.248 | 154 | -0.749 | 0.455 |
| Expertise | -0.1 | 0.28 | 1597 | -0.358 | 0.72 |
| Gender | -0.0646 | 0.118 | 73 | -0.547 | 0.586 |
| Genre | 0.0147 | 0.0512 | 73 | 0.287 | 0.775 |
| Age | -0.000731 | 0.0045 | 73 | -0.163 | 0.871 |
| Fam. Level × Numerosity | -0.0613 | 0.109 | 154 | -0.56 | 0.576 |
| Level × Expertise | -0.0296 | 0.106 | 5464 | -0.279 | 0.78 |
| Numerosity × Expertise | 0.213 | 0.097 | 5464 | 2.19 | 0.0285 |
| Fam. Level × Numerosity × Expertise | -0.0501 | 0.0427 | 5464 | -1.17 | 0.241 |

**Table 10.** GLMM results for Pleasantness. The main factors and their interactions with participants as random factors. Four background factors have been also included in the analysis although the interactions with the main experimental factors have not been included in the GLMM analysis.

| Factor | Estimate | Std. Error | df | t value | P value |
|---|---|---|---|---|---|
| Intercept | 3.13 | 0.69 | 292 | 4.53 | 8.48e-06 |
| Fam. Level | -0.0435 | 0.259 | 223 | -0.168 | 0.867 |
| Numerosity | 0.022 | 0.237 | 223 | 0.0929 | 0.926 |
| Expertise | 0.0301 | 0.298 | 2293 | 0.101 | 0.92 |
| Gender | 0.0612 | 0.118 | 75 | 0.518 | 0.606 |
| Genre | 0.0143 | 0.0581 | 75 | 0.246 | 0.807 |
| Age | 0.00212 | 0.00453 | 75 | 0.468 | 0.641 |
| Fam. Level × Numerosity | -0.0579 | 0.104 | 223 | -0.556 | 0.579 |
| Fam. Level × Expertise | -0.00274 | 0.116 | 5606 | -0.0237 | 0.981 |
| Numerosity × Expertise | 0.13 | 0.106 | 5606 | 1.23 | 0.217 |
| Fam. Level × Numerosity × Expertise | -0.0674 | 0.0466 | 5606 | -1.45 | 0.148 |

**Table 11.** GLMM results for Preference. The main factors and their interactions with participants as random factors. Four background factors have been also included in the analysis although the interactions with the main experimental factors have not been included in the GLMM analysis.

| Factor | Estimate | Std. Error | df | t value | P value |
|---|---|---|---|---|---|
| Intercept | 2.54 | 0.721 | 269 | 3.53 | 0.000496 |
| Level | -0.0148 | 0.272 | 204 | -0.0542 | 0.957 |
| Numerosity | 0.0137 | 0.249 | 204 | 0.0552 | 0.956 |
| Expertise | -0.0254 | 0.296 | 3444 | -0.0859 | 0.932 |
| Gender | -0.0955 | 0.0987 | 75 | -0.967 | 0.337 |
| Genre | 0.0204 | 0.0416 | 75 | 0.491 | 0.625 |
| Age | -0.00332 | 0.00474 | 75 | -0.7 | 0.486 |
| Fam. Level × Numerosity | 0.0987 | 0.11 | 204 | 0.901 | 0.369 |
| Fam. Level × Expertise | 0.0855 | 0.118 | 5606 | 0.723 | 0.47 |
| Numerosity × Expertise | -0.0349 | 0.108 | 5606 | -0.323 | 0.746 |
| Fam. Level × Numerosity × Expertise | 0.0227 | 0.0476 | 5606 | 0.476 | 0.634 |

**Table 12.** GLMM results for Tension. The main factors and their interactions with participants as random factors. Four background factors have been also included in the analysis although the interactions with the main experimental factors have not been included in the GLMM analysis.

| Factor | Estimate | Std. Error | df | t value | P value |
|---|---|---|---|---|---|
| Intercept | 4.11 | 0.808 | 193 | 5.09 | 8.56e-07 |
| Level | -0.43 | 0.321 | 159 | -1.34 | 0.182 |
| Numerosity | -0.31 | 0.293 | 159 | -1.06 | 0.292 |
| Expertise | -0.09 | 0.324 | 3247 | -0.278 | 0.781 |
| Gender | 0.111 | 0.101 | 69 | 1.1 | 0.275 |
| Genre | -0.00137 | 0.0467 | 69 | -0.0293 | 0.977 |
| Age | 0.00244 | 0.00397 | 69 | 0.615 | 0.541 |
| Fam. Level × Numerosity | 0.0601 | 0.129 | 159 | 0.465 | 0.642 |
| Fam. Level × Expertise | 0.139 | 0.13 | 5180 | 1.07 | 0.285 |
| Numerosity × Expertise | 0.212 | 0.118 | 5180 | 1.79 | 0.0733 |
| Fam. Level × Numerosity × Expertise | -0.138 | 0.0521 | 5180 | -2.65 | 0.00812 |

**Table 13.** GLMM results for Harmoniousness. The main factors and their interactions with participants as random factors. Four background factors have been also included in the analysis although the interactions with the main experimental factors have not been included in the GLMM analysis.
